# Mitochondrial DNA sequence variation in Finnish patients with matrilineal diabetes mellitus

**DOI:** 10.1186/1756-0500-5-350

**Published:** 2012-07-10

**Authors:** Heidi K Soini, Jukka S Moilanen, Saara Finnila, Kari Majamaa

**Affiliations:** 1 Institute of Clinical Medicine, Department of Neurology, University of Oulu, P.O. Box 5000, FI-90014, Oulu, Finland; 2 Clinical Research Center, Oulu University Hospital, University of Oulu, P.O. Box 5000, FI-90014, Oulu, Finland; 3 Institute of Clinical Medicine, Department of Clinical Genetics, University of Oulu, P.O. Box 5000, FI-90014, Oulu, Finland

**Keywords:** Mitochondrial DNA, diabetes, Mitochondrial DNA haplogroups, m.16189 T>C, Maternal inheritance

## Abstract

**Background:**

The genetic background of type 2 diabetes is complex involving contribution by both nuclear and mitochondrial genes. There is an excess of maternal inheritance in patients with type 2 diabetes and, furthermore, diabetes is a common symptom in patients with mutations in mitochondrial DNA (mtDNA). Polymorphisms in mtDNA have been reported to act as risk factors in several complex diseases.

**Findings:**

We examined the nucleotide variation in complete mtDNA sequences of 64 Finnish patients with matrilineal diabetes. We used conformation sensitive gel electrophoresis and sequencing to detect sequence variation. We analysed the pathogenic potential of nonsynonymous variants detected in the sequences and examined the role of the m.16189 T>C variant. Controls consisted of non-diabetic subjects ascertained in the same population. The frequency of mtDNA haplogroup V was 3-fold higher in patients with diabetes. Patients harboured many nonsynonymous mtDNA substitutions that were predicted to be possibly or probably damaging. Furthermore, a novel m.13762 T>G in *MTND5* leading to p.Ser476Ala and several rare mtDNA variants were found. Haplogroup H1b harbouring m.16189 T > C and m.3010 G > A was found to be more frequent in patients with diabetes than in controls.

**Conclusions:**

Mildly deleterious nonsynonymous mtDNA variants and rare population-specific haplotypes constitute genetic risk factors for maternally inherited diabetes.

## Background

Mitochondria play a key role in metabolism, heat production and apoptosis and contribute to aging and formation of reactive oxygen species (ROS) [1,2]. Above all, mitochondrial oxidative phosphorylation (OXPHOS) produces adenosine triphosphate (ATP) that is the energy form driving cellular processes. Both nuclear genome and mitochondrial DNA (mtDNA) code for the subunits of the respiratory chain complexes that catalyse the reactions of OXPHOS. Maternally inherited mtDNA consists of 16 569 base pairs and codes for 13 proteins of the respiratory chain, while the remaining more than 70 subunits are encoded by the nuclear genome. Furthermore, mtDNA encodes 2 ribosomal RNAs and 22 tRNAs required for mitochondrial protein synthesis.

MtDNA is more prone to mutations than nuclear DNA leading to variation that can be used as a tool in population genetics. Certain polymorphisms mark branch points in the phylogenetic tree of human mtDNA and define population-specific haplogroups. Europeans belong to mtDNA haplogroups H, V, U, K, T, J, I, W, X and Z [3]. Certain mtDNA haplogroups have been associated with susceptibility to various diseases but also with beneficial traits like longevity [4]. It has been postulated that certain mtDNA polymorphisms either decrease or increase the patency of the mitochondrial respiratory chain and the production of harmful ROS. Furthermore, some mildly harmful polymorphism can bring forth subtle changes in translation, replication or production of regulatory elements of mtDNA [4]. Supramolecular assembly of mitochondrial respiratory chain complexes have been suggested to create a dynamic supercomplex. Amino acid variation in subunits of complexes can have minor effects on the stability and assembly of the supercomplexes and may lead to impaired function of OXPHOS or increased ROS production [5,6].

Non-neutral patterns have been found in human mtDNA indicating that slightly deleterious mutations may be present in the population [7]. According to the neutral theory of molecular evolution, variation at the molecular level is due to the interaction between genetic drift and mutation, rather than being actively maintained by selection. Selection against mildly deleterious mtDNA mutations suggests that increased sequence variation could be a risk factor for complex diseases such as type 2 diabetes [8,9]. It has been proposed that recent bottleneck events can lead to the over-representation of minor mtDNA alleles in population and to the emergence of population-specific risk factors for diabetes [10].

Insulin-dependent type 1 and non-insulin dependent type 2 are the two main types of diabetes. Type 2 diabetes has an excess of maternal transmission and can be inherited through multiple maternal generations [11]. Mitochondrial diabetes constitutes the third type of diabetes. It accounts for 1 % of all diabetes cases and is often accompanied by hearing impairment and other multi-organ symptoms typical for mitochondrial diseases. Mitochondrial diabetes is most often caused by the m.3243A > G mutation in *MTTL*[11-13].

The m.16189 T > C polymorphism near the termination-associated segment in the non-coding region of mtDNA creates an uninterrupted cytosine tract. The tract varies in length between five and 13 consecutive cytosines, the wild type genome containing nine cytosines with an intervening thymine in position m.16189 after the fifth cytosine. The m.16189 T > C polymorphism diminishes the rate of mtDNA replication and causes a lower mtDNA copy number and a disadvantage in metabolic efficiency [14]. It has been speculated that m.16189 T acts as a brake for replication slippage or facilitates the whole process of replication. The m.16189 T > C polymorphism has been linked with maternally inherited thinness [15], thinness at birth [16] and increased body mass index [17], and increased frequency of type 2 diabetes in the UK [18] and in Asia [19,20].

In this study we have attempted to identify mtDNA polymorphisms or their combinations that could increase the risk of maternally inherited diabetes. For this purpose, we determined complete mtDNA sequences in 64 Finnish patients with maternally inherited diabetes and compared them to complete mtDNA sequences in 192 population controls.

Subjects and Samples

Patients were identified from the records of 40 out of 42 local-authority health care units in Northern Finland. The discharge diagnoses and the diabetes register of two hospitals were reviewed. The selected patients had started insulin treatment for diabetes between the ages of 20 and 45 years and had maternal first- or second-degree relatives with diabetes, hearing loss or epilepsy. A total of 175 patients were identified, 111 of them reported at least one first-degree maternal relative with diabetes. We received 82 blood samples.

We calculated a crude proportion of affected maternal relatives for each patient using N_affected_/N_total_[21] after exclusion of the probands. We then selected 64 patients with the highest calculated crude proportion of maternal relatives. The mean crude proportion for the selected patients was 0.30 (standard deviation 0.19; median 0.25; range 0.077 - 1). Restriction fragment analysis was used to verify the absence of m.3243A > G and m.8344A > G in the selected samples.

Population controls consisted of 480 healthy Finnish Red Cross blood donors from Northern Finland. It was required that the donor and his or her mother were born in the same region, did not have diabetes, sensorineural hearing impairment or any neurological ailments. MtDNA haplogroups have been determined in all the 480 controls [3], the hypervariable segment I in the D-loop has been sequenced in 403 controls [22] and the entire mtDNA sequence has been determined in 192 controls [3]. The ethics committee of the University of Oulu and the Finnish Red Cross have approved the study protocol. All participants signed a written informed consent for participation in the study, all participants were adults.

## Methods

### Conformation sensitive gel electrophoresis (CSGE)

Total DNA was extracted from blood using the QIAmp Blood Kit (Qiagen, Hilden, Germany). MtDNA haplogroups were determined by restriction fragment analysis and Conformation sensitive gel electrophoresis (CSGE) was performed as described previously [23-25]. MtDNA coding region spanning the nucleotides m.577-m.16090 was amplified in 63 partially overlapping fragments. PCR fragments were amplified in a total volume of 30 ?l in 30 cycles through denaturation at 94 °C for 1 min, annealing at a primer-specific temperature and extension at 72 °C for 1 min and a final extension for 10 min. A touchdown-PCR protocol was used in parallel yielding similar results. The mean size of the amplified fragments was 354 bp. Small volume (3–10 ?l) of the PCR product was used for heteroduplex formation. Each amplified fragment was mixed with a reference sample and denatured at 95 °C for 5 min and annealed at 68 °C for 30 min for heteroduplex formation. Samples were electrophoresed using 15 % polyacrylamide gel overnight at a constant voltage of 400 V in room temperature. After electrophoresis the gel was stained in 150?g/l of ethidium bromide for 5 min and destained in water. Finally, the gel was transferred to ultraviolet transilluminator and photographed (Grab-IT Annotating Grabber 2.04.7; UVP).

### Sequencing

PCR fragments that differed in mobility in CSGE were sequenced (ABI PRISM ™ 377 Sequencer using DYEnamic ET Terminator Cycle Sequencing Kit; Amersham Pharmacia Biotech Inc., Buckinghamshire, U.K.) after purification with exonuclease I and shrimp alkaline phosphatase [26]. The primers for sequencing were the same as those used for the amplification of the 63 CSGE fragments. The D-loop spanning nucleotides m.15975 – m.725 was sequenced directly.

### Analysis of substitutions

Sequences were compared to the revised Cambridge reference sequence [GenBank:NC_012920] [27] and to mtDNA sequences available in the GiiB-JST mtSNP database http://mtsnp.tmig.or.jp/mtsnp/index_e.shtml[28], mtDB Human Mitochondrial Genome database http://www.mtdb.igp.uu.se[29] and Mitomap http://www.mitomap.org[30] accessed in October 2011. All variants were also compared to our 192 population-specific controls [3]. Novel substitutions were confirmed by restriction fragment length polymorphism-method (RFLP) or sequencing in both directions at least twice from different PCR products. Previously reported pathogenic mutations were identified according to Mitomap. Base conservation in tRNA genes was determined using Mamit tRNA: Compilation of Mammalian mitochondrial tRNA genes http://mamit-tRNA.u-strasbg.fr[31] and conservation of other mtDNA-encoded genes using the GiiB-JST mtSAP evaluation http://mtsnp.tmig.or.jp/cgi-bin/mtsnp/specAlign/ctrlSpecAlignE.cgi[28].

PolyPhen-2 version 2.1.0 [32] was used for prediction of functional effects of nonsynonymous mutations on subunit proteins http://genetics.bwh.harvard.edu/pph2/. For each mutation PolyPhen-2 calculates a naïve Bayes posterior probability that this mutation is damaging. Mutations are then classified as benign if the calculated probability is less than 50 %, possibly damaging if the probability is greater than 50 % and probably damaging if the probability is greater than 90 %. HumDiv-model was used, because it is recommended for rare alleles in complex diseases and for mildly deleterious alleles [32]. The m.7444 G > A variant in haplogroup V creates a stop codon and, in consequence, could not be analyzed. PredictProtein was used to predict the secondary structure of subunits harbouring the variant amino acids identified among the patients [33]. Fisher’s exact two-tailed test was used to compare haplogroup frequencies, mutation frequencies and m.16189 T > C genotype frequencies. Phylogenetic networks of mtDNA sequences were based on the median algorithm [34].

## Findings

### Frequency of mtDNA haplogroups

**Figure 1:**
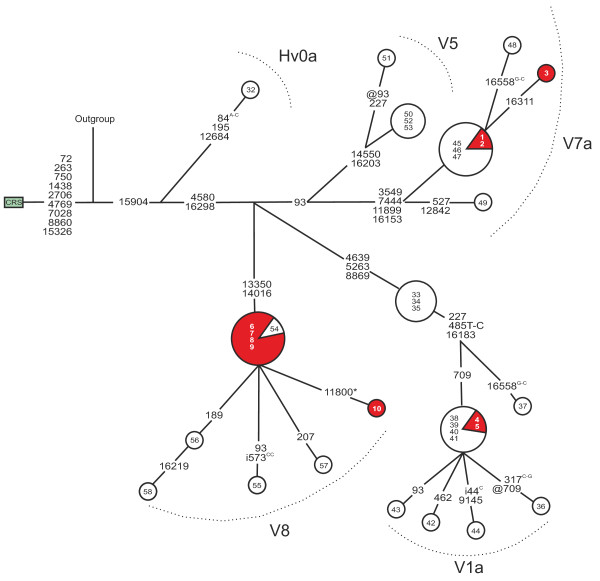
**Phylogenetic network of mtDNA haplogroup V in patients with diabetes and controls.** Red nodes, patients with diabetes (N = 10); white nodes, population controls (N = 27). Inside the nodes, cases identified by numbers 1–10; controls numbered as previously published [3]. Fast evolving sites m.303, m.311 and m.16519 were not included in the network. Outgroup, an African sequence [GenBank:AF346980]; CRS, the revised Cambridge Reference Sequence [GenBank:NC_012920]. Unless marked otherwise, mtDNA variants are transitions. Superscripts indicate transversions, inserted or deleted nucleotides: i = insertion, @ = back mutation, * = heteroplasmic mutation.

Haplogroup V was three times more common in patients with maternally inherited diabetes than in the population controls (*p* = 0.0066 for difference, Table 1). Subhaplogroup V8, defined by m.13350A>G and m.14016G>A, was found at a frequency of 7.7 % among the patients and 2.6 % among the controls (*p* = 0.13 for difference), whereas the frequency of subhaplogroup V1a was 3.1 % in patients with diabetes and 6.2 % in the controls (*p* = 0.53 for difference) (Figure 1). Interestingly, we discovered one patient belonging to haplogroup R1a, which is rare in the Finnish population.

**Table 1:** Frequencies of mtDNA haplogroups among 64 patients with maternally inherited diabetes and 480 controls

	**DM**		**Controls**^**1**^		
**Haplogroup**	**(N)**	**(%)**	**(N)**	**(%)**	***p*-value**
H	23	35.9	188	39.1	NS
U	15	23.4	134	27.9	NS
V	10	15.6	27	5.6	0.0066*
T	5	7.8	12	2.5	NS
I	3	4.6	15	3.1	NS
K	3	4.6	12	2.5	NS
W	2	3.1	46	9.5	NS
J	2	3.1	26	5.4	NS
Z	0	0	10	2.0	NS
X	0	0	7	1.4	NS
Other^2^	1	1.5	3	0.6	NS

^1^The controls are those from [3].

^2^One patient with diabetes belonged to haplogroup R.

*The frequency of haplogroup V differed from that of the population controls (*p* = 0.0066, Fisher’s exact two-tailed test, no adjustments were made for multiple comparisons).

### MtDNA variation in patients with maternally inherited diabetes mellitus

**Figure 2:**
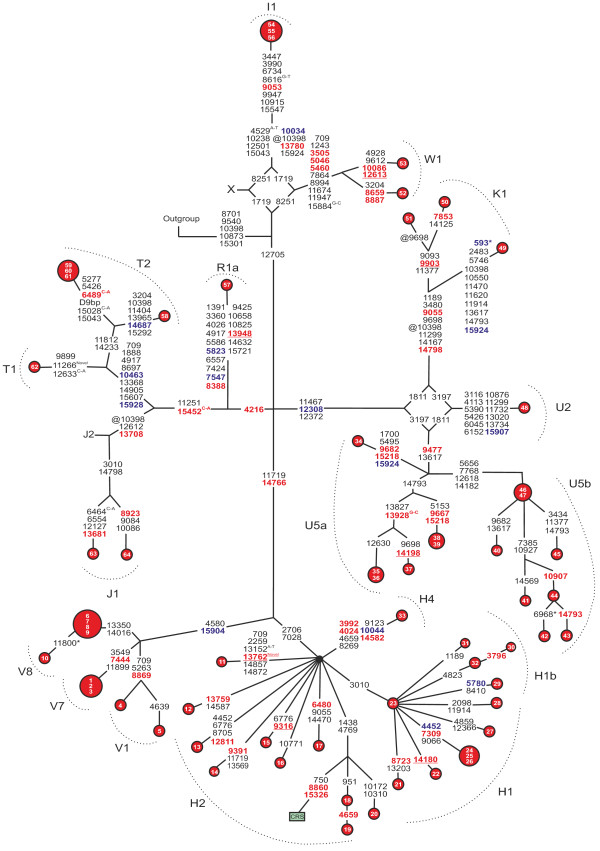
**Phylogenetic network based on the coding sequence of mtDNA from 64 patients with matrilineal diabetes.** Inside the nodes, cases identified by numbers. Fast evolving sites m.303, m.311 and m.16519 were not included in the network. Outgroup, an African sequence [GenBank:AF346980]; CRS, the revised Cambridge Reference Sequence [GenBank:NC_012920]. Superscripts indicate transversions, novel variants and inserted or deleted nucleotides: i = insertion, D = deletion, @ = back mutation, * = heteroplasmic mutation. D9bp, deletion spanning between the positions m.8281 and m.8289. Nonsynonymous substitutions are shown in red font, the tRNA variants in blue font. Underlined nonsynonymous variants were deemed to be possibly or probably damaging in PolyPhen-2 analysis.

**Figure 3:**
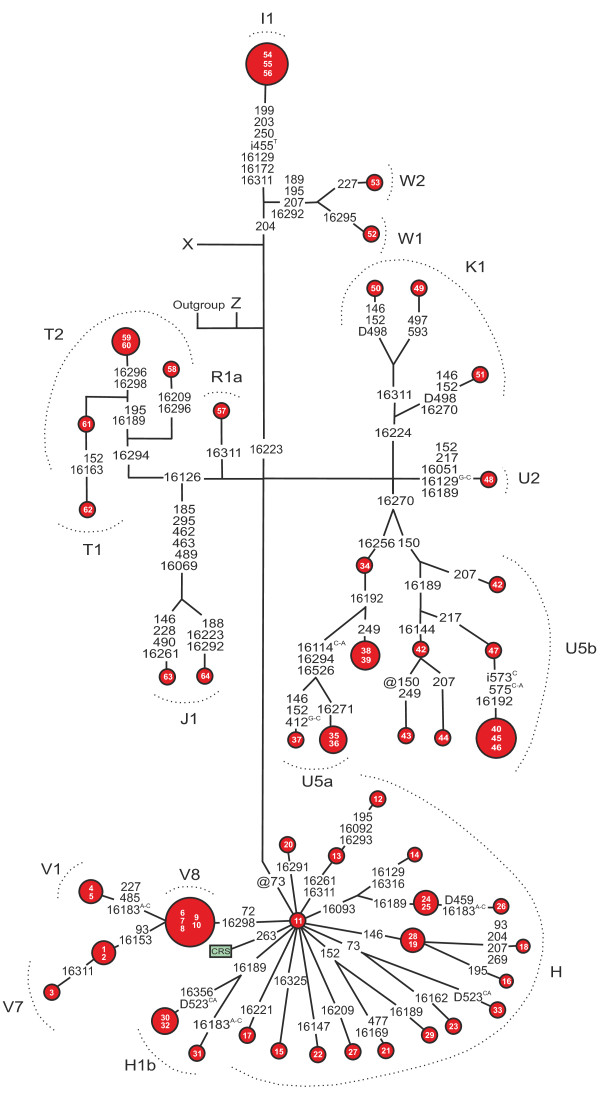
**Phylogenetic network based on the mtDNA D-loop sequences from 64 patients with matrilineal diabetes.** The fast evolving site m.16519 and the variants in the C-tract between the positions m.303-315 were not included in the network.The fast evolving site m.16519 and the variants in the C-tract between the positions m.303-315 were not included in the network. Outgroup, an African sequence [GenBank:AF346980]; CRS, the revised Cambridge Reference Sequence [GenBank:NC_012920]. Unless marked otherwise, the polymorphic variants are transitions. Superscripts indicate transversions, inserted or deleted nucleotides and novel variants: i = insertion, D = deletion, @ = back mutation.

A total of 209 coding region variants (Figure 2) and 83 D-loop variants (Figure 3) were found among patients with diabetes. Two of the variants were novel including the synonymous m.11266C>T transition in *MTND4* and the nonsynonymous m.13762T>G transversion in *MTND5* leading to p.Ser476Ala. Seven nonsynonymous variants were predicted to be possibly or probably damaging by PolyPhen-2 analysis (Table 2).

**Table 2:** Possibly or probably damaging nonsynonymous mtDNA variants predicted by PolyPhen-2

**Variant**	**Gene**	**Protein change**	**Probability (%)**^**1**^	**mtDB hits**^**2**^**(N)**	**Family history**
m.13762 T > G	*MTND5*	p.Ser476Ala	50.2	-	DM, DEM, HL
m.9316 T > C	*MTCO3*	p.Phe37Ser	74.7	1	DM
m.9903 T > C	*MTATP6*	p.Phe233Leu	75.6	1	DM, T
m.14180 T > C	*MTND6*	p.Tyr165Cys	90.5	18	DM, HL DEM
m.12613 G > A	*MTND5*	p.Ala93Thr	97.2	4	DM
m.14198 G > A	*MTND6*	p.Thr159Met	98.4	2	DM
m.13948 C > T	*MTND5*	p.Pro538Ser	99.4	2	DM, DEM, T

^1^ Probability indicates the probability of the variant being damaging as estimated by PolyPhen-2. Probabilities >50 % are classified as possibly damaging and probabilities >90 % are classified as probably damaging.

^2^ Number of sequences harbouring the variant among 1865 complete mtDNA sequences and 839 coding region sequences at http://www.mtdb.igp.uu.se/

DM, diabetes mellitus; DEM, dementia; HL, hearing loss; T, tremor.

Three previously reported pathogenic nonsynonymous mutations were found, including m.4659G>A in *MTND2* creating p.Ala64Thr in patient 19 [35], m.6480G>A *MTCO1* creating p.Val193Ile in patient 17 [36] and m.6489C>A in *MTCO1* creating p.Leu196Ile in patients 59, 60 and 61 [37]. The m.4659G>A mutation has been reported to contribute to Parkinson’s disease, m.6480G>A has been linked to prostate cancer and m.6489C>A to epilepsia partialis continua. However, all three variants were predicted to be benign in PolyPhen-2 analysis.

### MtDNA variants in tRNA encoding genes

We discovered 13 substitutions in genes encoding tRNAs among the patients (Figure 2). The variants were most abundant in *MTTT* encoding tRNA^Thr^, where four polymorphisms were found including m.15904C>T associated with haplogroup V, m.15907A>G associated with subhaplogroup U2, m.15924A>G that occurs in several haplogroups [38] and m.15928G>A associated with haplogroup T. Heteroplasmic m.593T>C was found in *MTTF* encoding tRNA^Phe^ in patient 49. Two mutations with previously reported disease associations were discovered in *MTTC* encoding tRNA^Cys^. Patient 29 belonging to subhaplogroup H1b harboured m.5780G>A that has been found in a patient with sensorineural hearing impairment [39] and patient 57 belonging to subhaplogroup R1a harboured m.5823A>G that has been found in a patient with motor neuron disease and temporal lobe epilepsy [40]. The remaining six substitutions in genes encoding tRNAs included m.7547T>C in *MTTD*, m.10034T>C and m.10044A>G in *MTTG*, m.10463T>C in *MTTR*, m.12308A>G in *MTTL2* and m.14687A>G in *MTTE*.

### m.16189T>C polymorphism in matrilineal diabetes mellitus patients

**Figure 4:**
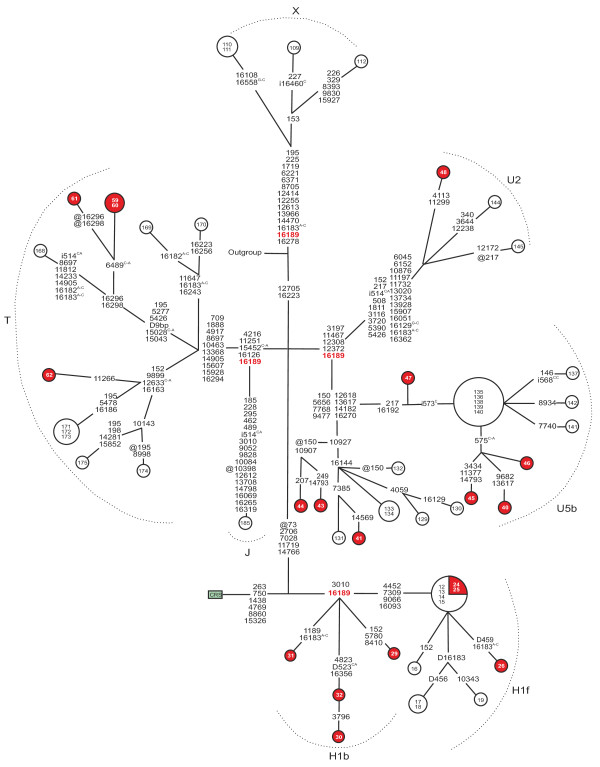
**Phylogenetic network of mtDNA harbouring m.16189 T > C.** Red nodes, patients with matrilineal diabetes (N = 19); white nodes, population controls (N = 37). Cases are numbered 24–62, controls numbered as previously published [3]. The fast evolving site m.16519 and the variants in the C-tract between the positions m.303-315 were not included in the network. Outgroup, an African sequence [GenBank:AF346980]; CRS, the revised Cambridge Reference Sequence [GenBank:NC_012920]. Unless marked otherwise, mtDNA variants are transitions. Superscripts indicate transversions, inserted or deleted nucleotides: i = insertion, D = deletion, @ = back mutation. D9bp, deletion spanning between the positions m.8281 and m.8289.

The frequency of m.16189T>C or the sequence of the cytosine tract surrounding this nucleotide did not differ between patients with diabetes and the controls (Table 3). Interestingly, phylogenetic analysis of the sequences harbouring m.16189T>C revealed four patients but no population controls belonging to subhaplogroup H1b (*p* = 0.0038 for difference; Figure 4). We then searched for similar sequences among the 1865 complete and 839 coding region mtDNA sequences deposited in the mtDB database and found 30 sequences that harboured m.16189T>C and m.3010G>A. Only three of them [GenBank:AY195775, GenBank:AY738975, GenBank:AY738982] belonged to subhaplogroup H1b, while the remaining sequences belonged to subhaplogroup H1f or to haplogroup J or D.

**Table 3:** Number of cytosines in the m.16179–m.16195 region in matrilineal diabetes patients and controls

**Cytosines**		**DM**		**Controls**	
**(N)**	**Sequence**	**(N)**	**(%)**	**(N)**	**(%)**
5*	CAAAACCCCCTCCCCAT	45	70.3	292	72.4
7	CAAAACCTCCCCCCCAT	0	0	4	0.9
8	CAAAACCCCCCCCTCAT	5	7.6	27	6.6
10	CAAAACCCCCCCCCCAT	11	16.9	59	14.6
10	CAAACCCCCCCCCCAT	0	0	3	0.7
11	CAAACCCCCCCCCCCAT	2	3	15	3.7
11	CAAAACCCCCCCCCCCAT	1	1.5	0	0
12	CAACCCCCCCCCCCCAT	0	0	3	0.7

* The wild-type mtDNA, revised Cambridge Reference Sequence [GenBank:NC_012920] contains 5 consecutive cytosines followed by a thymine and 4 additional cytosines in position m.16184-16193. DM, patients with matrilineal diabetes mellitus.

## Discussion

Haplogroup V, more specifically subhaplogroup V8, was more common among patients with maternally inherited diabetes than among population controls in Northern Finland. Interestingly, haplogroup V has recently been reported to increase the risk of renal failure in patients with type 2 diabetes and haplogroup cluster HV has been associated with retinopathy in these patients [8]. Unfortunately, no data on diabetes complications was available for our patients.

Haplogroup V is common amongst the Saami people of Northern Scandinavia and North-Western Russia. About 40 % of the Finnish Saami belongs to haplogroup V [22,35,36]. Certain mtDNA haplotypes considered to be Saami specific are found in the Finnish population suggesting a genetic admixture, which appears to be more pronounced in northern Finland. For example, the frequency of haplogroup V is 3 % in northern Finland, but only 1 % in Central Finland [22]. In the present study, patients and controls were ascertained from the same geographic area in order to minimise the bias caused by patients and controls originating from different populations.

One patient with diabetes belonged to haplogroup R1a, which is uncommon in Finland. In Europe, it occurs in the Kurdish population, Russia, Poland and the Caucasus area [37]. Our patient also harboured m.5823A > G in *MTTC* encoding tRNA^Cys^. The m.5823A > G variant has previously been reported in a haplogroup R1 sequence originating from India [37] and as a pathogenic mutation in a Caucasian patient with motor neuron disease and temporal lobe epilepsy. Unfortunately, no information about the haplogroup of this patient is available [38]. Our patient with diabetes and with m.5823A > G had reported first-degree relatives with diabetes, dementia, hearing loss or tremor.

The m.5823A > G mutation is located in the amino acid acceptor stem of tRNA^Cys^ and alters a nonconserved base. The mutation was homoplasmic both in our patient and in the two previous cases. In the amino acid acceptor stem of tRNA^Cys^ three polymorphisms and no pathogenic mutations have been found. In general, mutations in the tRNA acceptor stem affect the stability of the tRNA molecule and several of such mutations have been reported to cause mitochondrial disease phenotypes [39,40]. The nonconserved nature of the mutated nucleotide and the homoplasmic state suggests that m.5823A > G is a polymorphism associated with haplogroup R rather than a disease-causing mutation.

We discovered the homoplasmic m.5780 G>A. A mutation in MTTC in a patient with first- and second degree relatives with diabetes and a first-degree relative with dementia. The same mutation, but in a heteroplasmic state has previously been found in a Finnish patient with sensorineural hearing impairment [41]. Both patients belonged to subhaplogroup H1b. The frequency of this subhaplogroup was 3.1 % in the present patients, but subhaplogroup H1b was absent in the 192 controls. In MTTCC loop of the tRNA, five polymorphisms and six mutations with disease associations have been reported. Four of the mutations are associated with sensorineural hearing impairment or deafness, one with progressive dystonia and one with mitochondrial encephalopathy [31]. The nucleotide in position m.5780 codes for an invariant conserved cytosine located in the TCys molecule. The present patient with diabetes and the previous patient with sensorineural hearing impairment shared identical mtDNA coding region sequences with the variants m.5780 G>A and m.8410 C>T suggesting that m.5780 G>A is a rare polymorphism in subhaplogroup H1b.

PolyPhen-2 analysis of the nonsynonymous mtDNA variants suggested seven possibly or probably damaging mutations (Table 2). The variants m.13948 C > T, m.14198 G > A, m.12613 G > A and m.14180 T > C had the highest probabilities predicting that these mutations are damaging. The m.13948 C > T variant in *MTND5* leading to p.Pro538Ser had a probability of 99.5 %, which makes it most probably a deleterious missense mutation.

We discovered two novel mtDNA variants. One was synonymous m.11266 C > T and the other was nonsynonymous m.13762 T > C in *MTND5* leading to p.Ser476Ala. PolyPhen-2 analysis predicted that m.13762 T > C is possibly damaging, although the site is not particularly conserved (Table 2). PredictProtein analysis predicted that this amino acid is located in a loop structure. These analyses suggested that m.13762 T > C is probably a benign variant.

Three mutations with previously reported disease associations were found in our patients but not in the controls. The three mutations included m.4659 G > A leading to p.Ala64Thr [42], m.6480 G > A leading to p.Val193Ile [43] and m.6489 C > A leading to p.Leu196Ile [44]. These mutations were classified as benign in PolyPhen-2 analysis. Three sequences harbouring m.4659 G > A were detected in the mtDB database. These sequences belonged to haplogroups J, D and L, respectively, while our patient belonged to subhaplogroup H2 indicating that m.4659 G > A has arisen in multiple haplogroups. PolyPhen-2 analysis and phylogenetic comparison suggested that m.4659 G > A is a homoplasic polymorphism. The m.6480 G > A variant was detected in the mtDB database in six samples belonging to haplogroups I, T2b, HV, L2, L3 and R31. Our patient with m.6480 G > A belonged to haplogroup H. These findings suggest that m.6480 G > A is a homoplasic polymorphism. Three of our patients with diabetes and five sequences in the mtDB database harboured m.6489 C > A. All eight sequences belonged to subhaplogroup T2 confirming that m.6489 C > A is a polymorphism associated with this subhaplogroup [45].

We did not find differences in the frequency of m.16189 T > C between patients and controls, or in the variation of the polycytosine tract surrounding this position (Table 4). The m.16189 T > C variant has been linked to type 2 diabetes in Asians [19,20] and biochemical studies have revealed that the increased length of the cytosine tract is associated with a lower mtDNA copy number [14]. Furthermore, m.16189 T > C has been associated with reduced ponderal index at birth and reduced birth weight, but not with diabetes status [46,47].

Phylogenetic analysis of case and control mtDNA sequences containing m.16189 T > C revealed four patients but no controls belonging to subhaplogroup H1b (Figure 4). Subhaplogroup H1 is defined by m.3010 G > A [48]. Subhaplogroups H1b and H1f both harbour m.3010 G > A and m.16189 T > C, but H1f harbours additional polymorphisms at positions m.4452, m.7309, m.9066, and m.16093. The combination of m.3010 G > A and m.16189 T > C is also present in the Asian subhaplogroup D4b [28]. Interestingly, an association between type 2 diabetes and m.16189 T > C has been found in Asians [19,20] and subhaplogroup D4b has been linked with a significantly increased risk for type 2 diabetes in Korean men [49]. These findings suggest that m.3010 G > A and m.16189 T > C occurring in subhaplogroup H1b and in subhaplogroup D4b contribute to the risk of diabetes, but the same variants occurring in subhaplogroup H1f do not have such an effect. Our statistical analysis is limited by the small sample size and, furthermore, the results must be regarded as population-spesific. More samples are needed to better understand the link between maternally inherited diabetes and the suggested mildly deleterious mtDNA variants and haplogroups.

## Conclusions

We determined 64 complete mtDNA sequences of Finnish patients with matrilineal diabetes and discovered an excess of haplogroup V. Seven possibly or probably damaging nonsynonymous mtDNA variants were found. We also discovered four patients belonging to subhaplogroup H1b, which harbours m.16189 T > C and m.3010 G > A. The same combination exists in subhaplogroup D4b, which has previously been associated with type 2 diabetes in Korean men. The m.16189 T > C and m.3010 G > A occuring together without H1f variants create an infavourable combination of mtDNA variants and could predispose to matrilineal diabetes. We conclude that evolutionary recent nonsynonymous mtDNA variants and rare population-specific haplotypes constitute genetic risk factors for maternally inherited diabetes.

Availability of supporting data

The data sets supporting the results of this article are available in the [GenBank] repository, [GenBank:JX171077, GenBank:JX171078, GenBank:JX171079, GenBank:JX171080, GenBank:JX171081, GenBank:JX171082, GenBank:JX171083, GenBank:JX17084, GenBank:JX17085, GenBank:JX17086, GenBank:JX17087, GenBank:JX17088, GenBank:JX17089, GenBank:JX17090, GenBank:JX17091, GenBank:JX17092, GenBank:JX17093, GenBank:JX17094, GenBank:JX17095, GenBank:JX17096, GenBank:JX17097, GenBank:JX17098, GenBank:JX17099, GenBank:JX17100, GenBank:JX17101, GenBank:JX17102, GenBank:JX17103, GenBank:JX17104, GenBank:JX17105, GenBank:JX17106, GenBank:JX17107, GenBank:JX17108, GenBank:JX17109, GenBank:JX17110, GenBank:JX17111, GenBank:JX17112, GenBank:JX17113, GenBank:JX17114, GenBank:JX17115, GenBank:JX17116, GenBank:JX17117, GenBank:JX17118, GenBank:JX17119, GenBank:JX17120, GenBank:JX17121, GenBank:JX17122, GenBank:JX17123, GenBank:JX17124, GenBank:JX17125, GenBank:JX17126, GenBank:JX17127, GenBank:JX17128, GenBank:JX17129, GenBank:JX17130, GenBank:JX17131, GenBank:JX17132, GenBank:JX17133, GenBank:JX17134, GenBank:JX17135, GenBank:JX17136, GenBank:JX17137, GenBank:JX17138, GenBank:JX17139, GenBank:JX17140].

## Abbreviations

mtDNA, Mitochondrial DNA; ROS, Reactive Oxygen Species; CSGE, Conformation Sensitive Gel Electrophoresis; D-loop, Displacement loop - noncoding mitochondrial DNA control region; SNP, Single Nucleotide Polymorphism; RFLP, Restriction Fragment Lenght Polymorphism.

## Competing interests

The authors declare that they have no competing interests.

## Authors’ contributions

HKS: Carried out the conformation sensitive gel electrophoresis, sequencing, mtDNA verified novel substitutions, constructed the phylogenetic trees and wrote the first version of the manuscript. JSM: Reviewed patient registers to select patients with maternally inherited diabetes mellitus, collected and edited the mtDNA sequence data into sequence files, submitted mtDNA sequences, contributed to the study plan, data analysis and the manuscript writing. SF: Contributed to the study plan and data analysis. KM: Collected and selected the patients, planned the study protocol, reviewed the manuscript critically. All authors read and approved the manuscript.
